# Sodium pentosan polysulfate resulted in cartilage improvement in knee osteoarthritis - An open clinical trial-

**DOI:** 10.1186/1472-6904-10-7

**Published:** 2010-03-28

**Authors:** Kenji Kumagai, Susumu Shirabe, Noriaki Miyata, Masakazu Murata, Atsushi Yamauchi, Yasuhumi Kataoka, Masami Niwa

**Affiliations:** 1 Department of Orthopaedic Surgery, Graduate School of Biomedical Sciences, Nagasaki University, 1-7-1, Sakamoto, Nagasaki city, Nagasaki prefecture, 852-8501, Japan; 2 Regent and Vice President, Center for Health and Community Medicine, Nagasaki University, 1-14 Bunkyou, Nagasaki city, Nagasaki prefecture, 852-8521, Japan; 3 Department of Pharmaceutical Care and Health Sciences, Faculty of Pharmaceutical Sciences, Fukuoka University, 8-19-1, Nanakuma, Jonan-ku, Fukuoka city, 814-0180, Fukuoka prefecture, JAPAN; 4 Department of Pharmacology, Graduate School of Biomedical Sciences, Nagasaki University, 1-12-4, Sakamoto, Nagasaki city, Nagasaki prefecture, 852-8523, Japan

## Abstract

**Background:**

Pentosan polysulfate sodium (pentosan) is a semi-synthetic drug manufactured from beech-wood hemicellulose by sulfate esterification of the xylopyranose hydroxyl groups. From in vitro and animal model studies, pentosan has been proposed as a disease modifying osteoarthritis drug (DMOAD). The objective of this study was to assess the efficacy, safety, and patient satisfaction in patients with mild radiographic knee osteoarthritis (OA) findings and OA-associated symptoms and signs.

**Methods:**

Twenty patients were assessed clinically at Nagasaki University Hospital. The radiographic indications of OA were grade 1 to 3 using the Kellgren-Lawrence Grading System (K/L grade). Pentosan used in this study was manufactured and supplied in sterile injectable vials (100 mg/ml) by bene GmbH, Munich, Germany. The study was a single-center, open-label trial. Treatment consisted of 6 weekly subcutaneous injections (sc) of pentosan (2 mg/kg). Patients were clinically assessed at entry and 1 to 8, 11, 15, 24 & 52 weeks post treatment. The results were analyzed using one way ANOVA and Dunnett's method.

**Results:**

Hydrarthroses were reduced quickly in all cases. The clinical assessments, i.e., knee flexion, pain while walking, pain after climbing up and down stairs, etc, were improved significantly and these clinical improvements continued for almost one year. The dose used in this study affected the blood coagulation test, but was within safe levels. Slightly abnormal findings were noted in serum triglycerides.

**Conclusions:**

Pentosan treatment in twenty patients with mild knee OA seemed to provide improvements in clinical assessments and C2C level of cartilage metabolism.

**Trial Registration:**

UMIN Clinical Trials Registry (UMIN-CTR) UMIN000002790

## Background

Osteoarthritis (OA) is the most widespread joint disease affecting the elderly population [1]. Non-steroidal anti-inflammatory drugs (NSAIDs), supplements of chondroitin sulfate and/or glycosaminoglycans are prescribed as non-operative treatments. Recently, intraarticular injection of hyaluronic acid (HA) has become a common treatment. Within the last few decades, the concept of disease-modifying osteoarthritis drugs (DMOADs) has been explored as an alternative therapeutic treatment for OA.

From the results of previous in vitro and animal model studies, we have proposed that the spectrum of pharmacological activities exhibited by pentosan polysulfate sodium (pentosan) would qualify it as DMOADs. However, there is little human clinical evidence to support this proposition. The aim of this study is to assess the clinical effectiveness, functional outcome, safety, and patient satisfaction of a series of subcutaneous injections of pentosan in patients with symptomatic primary OA of the knee. Parts of this study were presented at the International Society of Orthopedic Surgery and Traumatology (SICOT), in Hong Kong, China in 2008 and at the OsteoArthritis Research Society International (OARSI) World Congress on Osteoarthritis, in Rome, Italy in 2008.

## Methods

### 1. Study design

The clinical study was designed as a single-center, open-label trial with an 8-week treatment phase. The radiographic indications of OA were mild to moderate OA, as described below. The follow-up continued for twelve months.

### 2. Patient recruitment

Symptomatic patients with primary OA of the knee were consulted by senior orthopedic surgeons who discussed their preferred management strategy. The radiographic indications of OA were grades 1 to 3 using the Kellgren-Lawrence Grading System (K/L grade)[2].

The elimination of patients was carried out according to the following:

a. Patients who received previous intra-articular corticosteroid or another drug injection in the knee joint within the previous 3 months.

b. Patients who had other lower-extremity musculoskeletal disability or pain.

c. Patients who had pain exceeding 45 mm on a 100-mm visual analogue scale (VAS, 0- 100, 100 as worst pain) immediately following walking for 50 m.

d. Patients who had any bleeding tendency with anti-coagulant drugs (aspirin by way of exception) having gastric or duodenal ulcer or with suspicion of alimentary tract bleeding.

e. Patients who had other severe disease or handicap (for example, diseases involving the liver, kidney, and bone marrow).

f. Patients who had a past history of drug allergy.

g. Patients who were pregnant or were breastfeeding.

h. Patients who had difficulty providing us with information.

i. Patients who had difficulty with the informed consent.

About using NSAIDs, patients were not eliminated if they underwent a two-week wash-out period before entering into this study.

According to the above conditions, twenty patients with knee OA were recruited.

The patient characteristics were as follows:

The average age was 63 years (from 35 to 80). All cases were female. Eight cases had right knee OA, 12 had left knee OA. The classifications of the weight bearing radiographs in K/L grade were grade 2 in 18 cases, and grade 3 in 2 cases. The average WOMAC score at the first visit was 37.0 (from 18 to 70).

### 3. Utilized agents

Pentosan polysulfate SP 54, used as pentosan in this study, was manufactured and supplied in sterile injectable vials (100 mg/ml) by bene-Arzneimittel GmbH, Munich, Germany.

It was illegal to use pentosan in Japan according to the relevant Japanese laws. So the study was approved by the review board of the Graduate School of Biomedical Sciences, Nagasaki University.

### 4. Study Methods

Following consent, the treatment consisted of 6 weekly subcutaneous injections (sc) of pentosan (2 mg/kg), following the two weeks of test injections (the first was 25 mg, the second was 50 mg).

No aspiration was performed, even if synovial fluid was present in the knee. After the initial phase, all patients were advised to avoid NSAIDs for 52 weeks. Paracetamol (<2000 mg/day) was allowed for break-through pain. All patients were allowed to maintain standard physical therapy.

### 5. Outcome assessment

All patients were prospectively reviewed at entry and at weeks 1, 2, 3, 4, 8, 12, 16, 24, and 52 with initial question of pain at rest and walking, a physical examination of the knee and VAS for pain with ROM exercises, 50 m walking, walking up and down stairs, or at 5 minutes rest after exercises. To check the change of the metabolism in the cartilage, degradation of type II collagen (C2C) in the blood was measured with a commercial ELISA kit, in addition to the usual biochemical tests. Weight bearing radiographs were reviewed at baseline and at the end of study to grade the degree of OA using K/L grade. WOMAC 3.1 (Likert) was used to measure secondary effectiveness.

### 6. Adverse events

Safety was assessed at each visit. Pentosan is a heparin-like agent and is not used in Japan. Activated clotting time (ACT) is a measure of the anticoagulation affects of heparin. So we checked the level of ACT, as well as the usual blood coagulation test one hour after every injection. All adverse events (AE), however minor, were recorded.

### 7. Statistics

All data was handled by members who did not treat or check the patients. The data was compared between the entry point and each of the follow-up points. The statistical significance, compared with the value at entry, was determined by one-way analysis of variance(ANOVA)and Dunnett's method. A p value of < 0.05 was considered significant for all statistical tests. Comparison of data was performed on a personal computer using StatView for Windows, ver. 5.0.1; SAS Institute Inc, Cary, NC, U.S.A.

## Results

The trial began enrolling patients in November 2005 and finished in August 2007. All twenty patients were followed up for one year.

### 1. Primary outcome measurements

**Figure 1:**
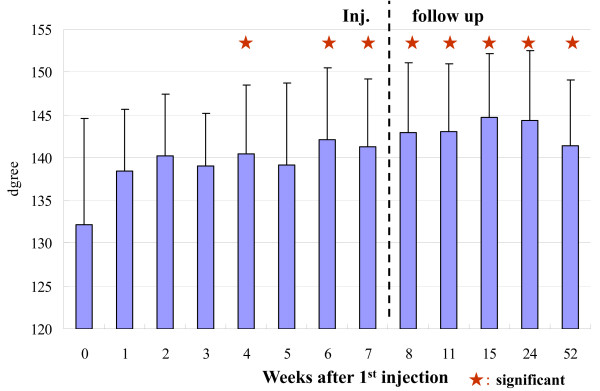
**Flexion angle of knee joint**. The hydrarthroses were reduced quickly in all cases. The ROM of knee joint was improved significantly. Clinical improvements were maintained at the one-year follow-up.

**Figure 2:**
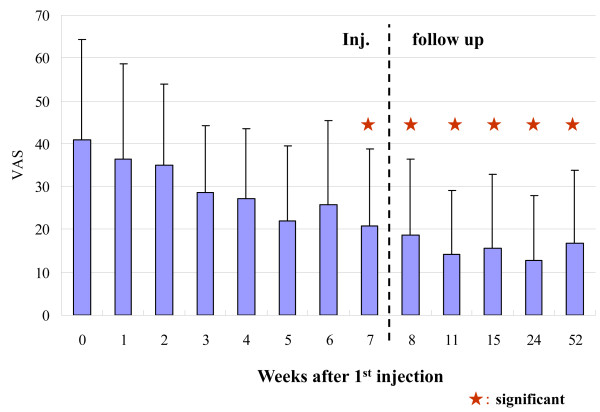
**Pain scores after climbing up and down stairs**. Pain scores after climbing up and down stairs, measured by VAS, was improved significantly. This improvement was maintained for almost one year.

**Figure 3:**
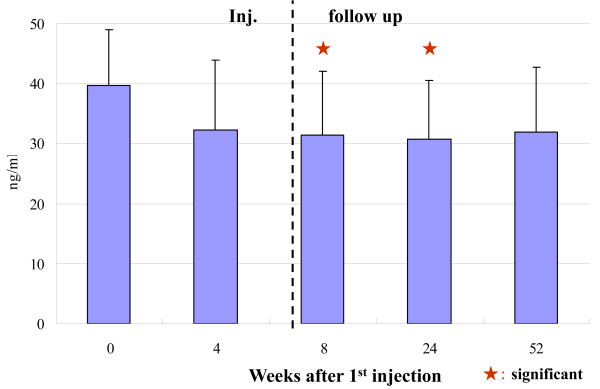
**Concentration of C2C in Blood**. The concentration of C2C in the blood decreased significantly at 8 and 24 weeks. To check the change of type II collagen metabolism, we measured C2C as a degradation marker of type II collagen, as C2C is said to be the only reliable marker of cartilage metabolism. The significant decreasing tendency of C2C is thought to be more objective than those parameters measured with VAS and ROM findings in this study.

The hydrarthroses were reduced quickly in all cases. The ROM of the knee joint improved significantly (Figure 1, Table 1). The clinical assessments, i.e., knee flexion, pain while walking, pain after climbing up and down stairs (Figure 2, Table 1), and pain just after ROM exercise improved significantly (table 1). The concentration of C2C in the blood decreased significantly (Figure 3, Table 1). The clinical benefits of this study continued for almost one year. In the X-ray findings, neither improvement nor degeneration were detected.

**Table 1:** Primary outcomes

	weeks	0	4	8	11	24	52
flexion angle	Ave ± SD	132.2 ± 12.4	140.5 ± 8.03	142.9 ± 8.25	144.75 ± 7.41	144.35 ± 8.11	141.4 ± 7.74
	% variation	0	5.8	7.61	9	8.7	6.5
	*: p ‹ 0.05		*	*	*	*	*
							
VAS after 50 m walking	Ave ± SD	27.7 ± 21.7	23.4 ± 16.4	16.5 ± 17.7	9.7 ± 14.3	9.8 ± 12.1	8.7 ± 12.2
	% variation	0	-30.49	-53.5	-65.0	-66.67	-64.65
	*: p ‹ 0.05						
							
VAS after 5 min rest	Ave ± SD	21.9 ± 21.3	18.8 ± 17.9	13.4 ± 16.1	8.8 ± 13.1	8.2 ± 13.7	8.1 ± 11.7
	% variation	0	-14.4	-38.6	-60	-62.6	-63
	*: p ‹ 0.05						
							
VAS up & down stair walking	Ave ± SD	41 ± 23.3	27.3 ± 16.2	18.6 ± 17.9	15.6 ± 17.2	12.8 ± 15.2	16.7 ± 17
	% variation	0	-33.5	-54.6	-62	-68.7	-59.3
	*: p ‹ 0.05			*	*	*	*
							
VAS ROM exercise	Ave ± SD	42.1 ± 25.4	29.3 ± 16.8	19.6 ± 17.8	13.0 ± 14.2	14.1 ± 15.8	14.9 ± 13.4
	%variation	0	-30.49	-53.5	-69.3	-66.67	-64.65
	*: p ‹ 0.05			*	*	*	*
							
C2C	Ave ± SD	39.7 ± 9.3	32.3 ± 11.7	31.4 ± 10.7		30.8 ± 9.8	31.9 ± 10.9
	%variation	0	-18.64	-20.91		-22.41	-19.65
	*: p ‹ 0.05			*		*	

The parameters were shown at entry point, 4^th^, 8^th^, 11^th^, 24^th^, and 52^nd ^weeks. They were provided as absolute levels, percentage of variation with time and statistical significance. There were statistically significant improvements from the baseline score in flexion angle at 4^th^, 8^th^, 11^th^, 24^th^, and 52^nd ^weeks; in VAS up and down stair walking and in VAS knee R.O.M. exercises at 8^th^, 11^th^, 24^th^, and 52^nd ^weeks; and in C2C concentration in blood at 11^th ^and 24^th ^weeks. The parameters, not limited to significant value, showed improvement in the percentage of variation with time. The improvement might appear in the late phase of injection and continue to 52 weeks.

The asterisks indicate statistically significant improvement.

### 2. Secondary outcomes measurements

**Figure 4:**
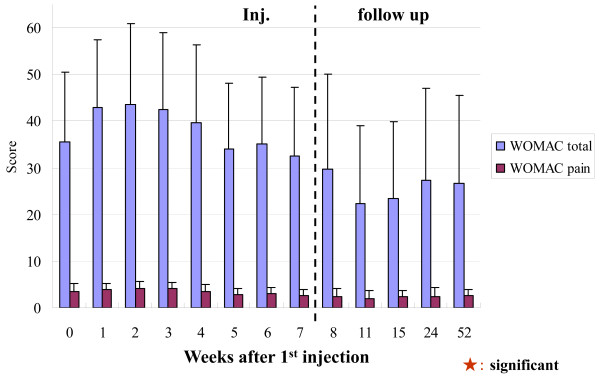
**WOMAC scores**. There were significant differences in the values between individual follow-up points in the WOMAC scores, but there was no significant difference in the data comparing the baseline and each follow-up. This may have been due to a problem with the baseline evaluation or the techniques of the score system

There was no statistical improvement in the total score of the WOMAC compared to the baseline measurements (Table 2). Both the WOMAC and the clinical assessments showed the same tendencies in time course curve (Figure 4). Pain while walking, as initial checks of each visit at our clinic, showed statistical improvement compared to the baseline measurements (Table 2).

**Table 2:** Secondary Outcomes

	weeks	0	4	8	11	24	52
WOMAC total	Ave ± SD	35.53 ± 15.03	39.75 ± 16.69	29.65 ± 20.4	23.4 ± 16.53	27.25 ± 19.68	26.74 ± 18.7
	% variation	0	11.88	-16.55	-34.14	-23.3	-24.75
	*: p ‹ 0.05						
							
WOMAC pain	Ave ± SD	3.47 ± 1.77	3.5 ± 1.47	2.45 ± 1.7	2.3 ± 1.3	2.4 ± 1.85	2.53 ± 1.43
	% variation	0	0.95	-29.33	-33.66	-30.78	-27.13
	*: p ‹ 0.05						
							
Pain at rest	Ave ± SD	14.85 ± 12.4	15.15 ± 8.03	14.15 ± 8.25	7.25 ± 7.41	6.85 ± 8.11	6.65 ± 7.74
	% variation	0	2.02	-4.71	-51.18	-53.87	-55.22
	*: p ‹ 0.05						
							
Pain while walking	Ave ± SD	32.1 ± 12.4	21.9 ± 8.03	18.5 ± 8.25	11.5 ± 7.41	11.8 ± 8.11	11.4 ± 7.74
	% variation	0	-31.8	-42.5	-64.3	-63.4	-64.5
	*: p ‹ 0.05				*	*	*

The parameters were shown at entry point, 4^th^, 8^th^, 11^th^, 24^th^, and 52^nd ^weeks. They were provided as absolute levels, percentage of variation with time and statistical significance. The asterisks indicate statistically significant improvement.

There were significant differences in the values between individual follow-up points in the WOMAC scores, but there was no significant difference in the data comparing the baseline and each follow-up. Pain when walking, as initial checks of each visit at our clinic, showed statistical improvement compared to the baseline measurements at 11^th^, 24^th^, and 52^nd^ weeks. These values showed remarkable improvement in percentage of variation with time.

### 3. Adverse events

**Figure 5:**
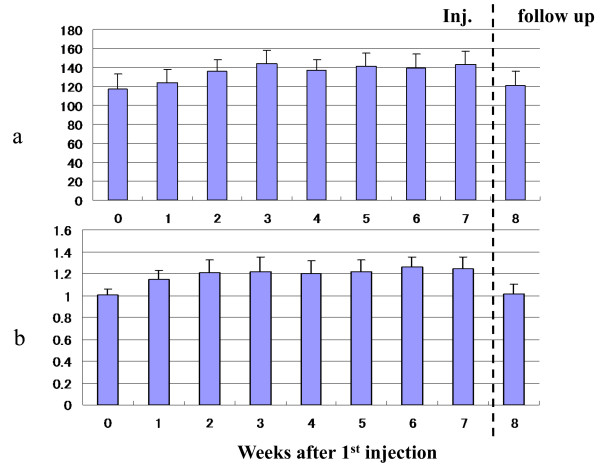
**Activated Clotting Time (ACT) and Prothrombin Time (INR) 1 hour after injection**. a: ACT, b: PT (INR). Activated clotting time (ACT) is a measure of the anticoagulation affects of heparin. So we checked the level of ACT, as well as usual blood coagulation test one hour after every injection. The dose of pentosan affected the ACT value, but the values were within a safe range. For PT (INR), the highest value was 1.47, which was weaker than the prophylactic administration of cardiovascular event.

**Figure 6:**
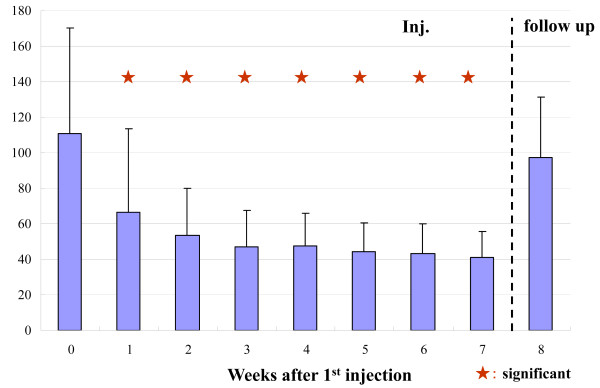
**Serum triglycerides 1 hour after injection**. Slightly abnormal findings were noted in serum triglycerides at one hour after injection, but this was reduced quickly in the follow-up period.

The dose of pentosan affected the blood coagulation tests, but the values were within a safe range. For aPPT, the highest value was 61.7. For PT (INR), the highest value was 1.47, which was weaker than the prophylactic administration of cardiovascular event. The value of ACT had the same tendency (Figure 5). Slightly abnormal findings were noted in serum chemistry: i.e., serum triglycerides at one hour after injection, but these were reduced quickly in the follow-up period (Figure 6).

Six patients had a small amount of subcutaneous bleeding at the injection site. Four patients claimed severe pain at injection, but the pain decreased rapidly. No patient suffered any major AE occurrence.

## Discussion

OA is the most widespread joint disease affecting the elderly population [1]. OA, by far the most common form of arthritis, has a growing impact on health care. Even now, there is no therapeutic agent that works directly on OA. Various treatment guidelines published by the American Academy of Orthopaedic Surgeons and American College of Rheumatology recommend viscosupplementation injections early in the treatment paradigm, before and during non-selective NSAIDs and cyclooxygenase-2 (COX-2) inhibitor therapy when those therapies are contraindicated, are ineffective, or cause adverse events[3-5]. Several drugs are candidates for DMOADs. Kahan et al. recommended chondroitins 4 and 6 sulfate according to their international, randomized, double-blind, placebo-controlled trial [6]. Henrotin recommended PIASCLEDINE^®^300 (Laboratories Expansciences, Courbevoie, France) among avocado/soybean unsaponifiables for his in vitro study and three well-conducted, randomized, placebo-controlled, trials [7,8]. However, further studies examining the method of action of these compounds is needed[9].

Pentosan is a semi-synthetic drug manufactured from European beech-wood hemicellulose by sulfate esterification. Its molecular weight is from 4000 to 6000 Daltons, and the average is 5700 Daltons. It was developed as a heparin-like agent, which interferes with the binding of factor Xa to thrombin by an AT-III-independent mechanism and has been used in Europe for conditions such as thrombosis prophylaxis and the treatment of phlebitis, for about fifty years. Pentosan is a very safe drug and is the first and only oral medication that has been approved by the US Food and Drug Administration for treating the pain or discomfort of interstitial cystitis [10,11]. According to Ghosh, the functional mechanism of pentosan for OA is as follows. In the cartilage, pentosan reduces cartilage degradation by directly and indirectly affecting inflammatory mediators such as MMP-3, IL-1 and TNF-alpha. Pentosan increased the amount of proteoglycan incorporated into the extracellular matrix. In the synovium, pentosan increases both the synthesis and the molecular weight of hyaluronan. In addition, pentosan has anti-inflammatory function and strong fibrinolytic activity, which improves the blood flow not only in the synovium but also in the subchondral bone [12] In the veterinary field from 1996 to date, there have been many studies and reports about the use of pentosan for OA [13,14]. Pentosan is now used worldwide. Ghosh and the Belgian study group conducted four small human studies in 1990s for OA of the knee, hip and fingers. In these studies, styles of medication were one per os, one intraarticular injection and two intramuscular injection. There was significant improvement in pain, mobility and synovial fluid condition[15]. The total dosage of pentosan in this study was planned as 12 mg/kg according to Ghosh's comments and his reports [12,15].

To check the change of type II collagen metabolism, we measured C2C as a degradation marker of type II collagen, as C2C is said to be the only reliable marker of cartilage metabolism[16]. The significant decreasing tendency of C2C is thought to be more objective than those parameters measured with VAS and ROM findings in this study.

The symptoms and signs of early stage, i.e., improvement of R.O.M., alleviation of hydrarthrosis and pain in walking, could be attributed to the synovial improvement by the anti-inflammatory action of pentosan. The cartilage metabolism can be measured accurately, in vivo, only by the degradation of collagen type II. In recent years, it has been reported that pentosan interacts with a disintegrin and metalloproteinase with thrombospondin motifs (ADAMTS)-4 and ADAMTS-5, and pentosan increased tissue inhibitor of metalloproteinase-3(TIMP-3)[17-19]. The above improvements of aggrecan might have occurred in this study and contributed to the long-term improvement.

The pentosan study by Ghosh and many HA studies had good WOMAC results [15,20-22]. In this study, there were significant differences in the values between individual follow-up points in the WOMAC scores, but there was no significant difference in the data comparing the baseline and each follow-up. This may have been due to a problem with the baseline evaluation or the techniques of the score system.

Lyon Schuss knee radiographs with definition of adequate alignment of the medial tibial plateau might be useful, as no improvement in X-ray findings could be detected in this study[23]. In describing a recently developed MRI technique, Eckstein reported that the quantitative magnetic resonance imaging (qMRI) of cartilage morphology is a promising tool for DMOAD development [24].

Also, the intraarthotic injection of hyaluronic acid is said to have been proven safe and effective for OA. But Raman reported that the incidence of local- and treatment-related AE vary from l% to 8% [25]. As pentosan is a subcutaneous injection administered far outside the joint, while hyaluronic acid requires intraarticular injection, pentosan is considerably safer in this respect. Furthermore, there are only very rare incidences of pentosan AE according to the complete reference for the Summary of Product Characteristics of Pentosan polysulfate SP 54 [26]. Treatment with pentosan seems to be considerably safer.

People rarely vary in their sensitivity to drugs, especially among Asian ethnicities, for example, gefitinib tablets, for advanced non-small cell lung cancer, and leflunomide, as DMARDs [27,28]. It is extremely important that careful administration and observation be carried out for new drugs.

## Conclusions

This clinical treatment with pentosan for twenty knee OA patients was considered as safe with only minor AE. Improved clinical assessments were observed and degradation of collagen type II was prevented with pentosan treatment.

## Competing interests

The authors declare that they have no competing interests.

## Authors' contributions

NM, MM and KK have made substantial contributions to conception and design, or acquisition of data, or analysis and interpretation of data. AY and YK participated in the design of the study, carried out the immunoassays, and performed the statistical analysis. SS and MN conceived of the study, and participated in its design and coordination and helped to draft the manuscript. KK, YK and MN have given final approval of the version to be published. All authors read and approved the final manuscript.

## Pre-publication history

The pre-publication history for this paper can be accessed here:

http://www.biomedcentral.com/1472-6904/10/7/prepub
